# Hematocolpos: Months-Long Mystery Revealed by Point-of-Care Ultrasound

**DOI:** 10.7759/cureus.83808

**Published:** 2025-05-09

**Authors:** Clara H Kraft, Justine Pagenhardt, Nick Denne, Joseph Minardi

**Affiliations:** 1 Emergency Medicine, West Virginia University School of Medicine, Morgantown, USA; 2 Emergency Medicine, West Virginia University, Morgantown, USA; 3 Emergency Medicine, West Viginia University, Morgantown, USA

**Keywords:** delayed diagnosis, hematocolpos, pediatric pain abdomen, pocus (point-of-care ultrasound), ultrasound in emergency medicine

## Abstract

A premenarchal female suffered recurrent abdominal pain for months before presenting to a critical access emergency department (ED), where point-of-care ultrasound (POCUS) finally revealed the underlying diagnosis of hematocolpos associated with imperforate hymen. Imperforate hymen, while rare, can lead to occlusion of the vaginal orifice during menarche, resulting in hematocolpos. Patients with hematocolpos can present with abdominal, low back, or pelvic pain, and delayed diagnosis may result in additional signs and symptoms, including tenesmus, constipation, urinary retention, and a palpable abdominal mass. Due to many potential etiologies of pediatric abdominal pain, attention to history of present illness and physical examination is critical to elucidate uncommon clinical diagnoses. POCUS is a safe, rapid, low-cost modality to evaluate the broad differential in pediatric abdominal pain. Early incorporation of POCUS may decrease diagnostic errors and patient morbidity associated with a delayed or inaccurate diagnosis.

## Introduction

Imperforate hymen, while rare, can lead to occlusion of the vaginal orifice during menarche, resulting in hematocolpos. Patients with hematocolpos can present with abdominal, low back, or pelvic pain, and delayed diagnosis may result in additional signs and symptoms, including tenesmus, constipation, urinary retention, and a palpable abdominal mass.

The differential diagnosis for pediatric abdominal pain is broad. Point-of-care ultrasound (POCUS) is a valuable tool in diagnosing multiple etiologies of abdominal pain. Ultrasound avoids ionizing radiation and can identify broad pathology such as appendicitis, intussusception, renal disease, hepatobiliary disease, as well as gynecologic conditions [1]. If appendicitis is suspected, both the American College of Radiology and the American College of Emergency Physicians support a diagnostic approach utilizing ultrasound prior to other imaging modalities [2,3]. POCUS can also help in the diagnosis of associated complications, such as urinary retention, and can identify hydronephrosis [4]. 

Overall, early utilization of POCUS in patients with abdominal pain shortens the differential diagnoses, expedites an accurate diagnosis, and decreases length of stay in the ED as well as length of stay in pediatric appendicitis [5-7]. A framework of widely available, competent application of POCUS in primary care and emergency clinical settings has the potential to shorten the time to diagnosis, decrease diagnostic errors, and decrease morbidity and mortality in this, as well as other less common causes of pediatric abdominal pain [5-7].

This case report was previously presented as a poster at the American Institute of Ultrasound in Medicine Conference, held on March 12-16, 2022.

## Case presentation

**Figure 1:**
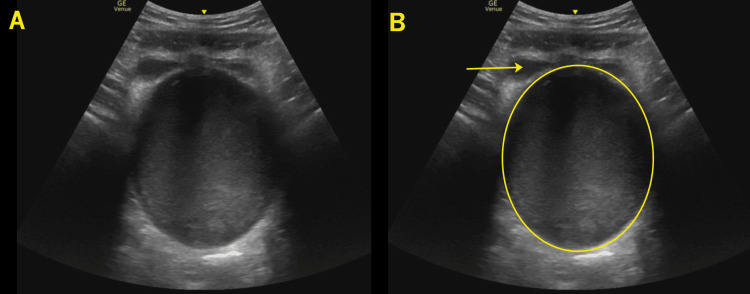
Transverse pelvis In this transverse image of the female pelvis, the decompressed bladder is noted anteriorly (frame B arrow) with a large echogenic fluid collection in the lower uterine segment and vaginal canal (frame B circle) representing retained menstrual blood.

**Figure 2:**
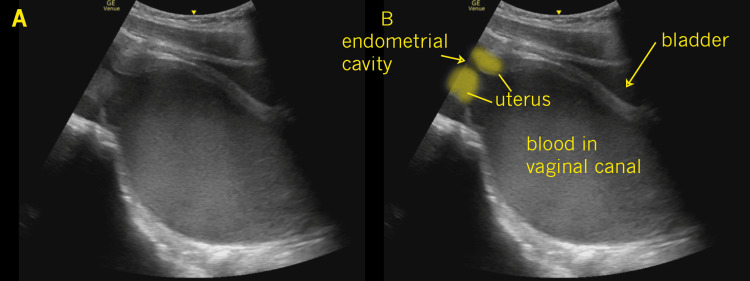
Longitudinal pelvis In this longitudinal image of the female pelvis, the bladder (labeled in frame B) is noted anterior to a large echogenic fluid collection within the lower uterine segment and vaginal canal (labeled in frame B). The lower borders of the uterus and the endometrial canal are noted on the left screen (labeled in frame B).

A 10-year-old premenarchal female presented to a critical access emergency department (ED) with a three-month history of lower abdominal pain. She had multiple previous evaluations, including three pediatric visits, one urgent care visit, and one ED visit, and was being treated for presumed constipation with oral laxatives and acetaminophen with no relief. She also underwent treatment for a urinary tract infection with a negative resultant culture. During prior evaluations, the patient underwent two separate abdominal X-ray examinations, demonstrating a “moderate colonic stool burden.” She described the pain as crampy, intermittent, and gradually worsening pain located in the infraumbilical region. There was no trauma, and no chronic medical problems. She had no fever. The abdominal exam showed no peritoneal signs, palpable mass, or other significant findings. A repeated abdominal X-ray was unremarkable. After evaluation by the advanced practice provider, the ED staff physician evaluated the patient concomitantly to assist with diagnosis. The physician performed a transabdominal POCUS study to evaluate alternative etiologies of the patient’s symptoms, given the lack of response to current treatment modalities. POCUS revealed unremarkable kidneys and gallbladder. In the lower abdomen, the uterus was noted to be enlarged and contained a large echogenic fluid collection predominantly in the lower segment extending into the vaginal canal (as seen in Figures 1, 2), measuring approximately 8 × 9 × 12 cm.

The right ovary appeared grossly normal, and the left ovary appeared enlarged with increased echogenicity (not shown). These findings prompted a genital examination revealing Tanner Stage V genitals with partially fused labia and a purple-colored mass at the vaginal introitus. Following genital examination, further history of fused vaginal labia was recalled by the patient’s family. The patient was referred to gynecology, where examination under anesthesia revealed an imperforate hymen with vaginal adhesions. A surgical hymenectomy was performed with improved symptoms at follow-up.

## Discussion

Imperforate hymen, while the most common female genital tract malformation, is relatively rare, with an estimated prevalence of between 1:1000 and 1:16000 [8,9]. When imperforate hymen is present, menarche results in blood collecting in the vagina, leading to vaginal dilation. As menstrual blood continues to collect, the uterus can become distended with blood. Further progression can result in hematosalpinx and even ruptured hematosalpinx and hemoperitoneum, and early diagnosis can help avoid the need for abdominal surgery [3].

Hematocolpos typically presents at puberty. Symptoms can be present for months or even years before a diagnosis is made [10,11]. Associated symptoms can include urinary retention and constipation, likely due to obstructive physiology [4,9,12,13]. The diagnosis is typically made by the physician based on physical exam findings and imaging, and while computed tomography and magnetic resonance imaging may be informative, ultrasound remains the diagnostic imaging modality of choice [10,14,15]. Regarding ultrasound technique, abdominal and pelvic structures can be visualized using a low-frequency curvilinear transducer. Placing the transducer superior to the pubic symphysis in a transverse view, the bladder should be identified as a landmark with the uterus and female pelvic anatomy posterior to the bladder. From here, the angle of the transducer should be adjusted to evaluate the adnexal structures and then rotated to obtain longitudinal views.

In evaluating patients with nonspecific abdominal pain, we suggest a syndromic approach focused on the differential diagnosis derived from a thorough history and physical. Syndromic ultrasound is performing ultrasound guided by the clinical syndrome and the associated differential diagnosis in regard to that syndrome, rather than focusing on a single particular organ system. We believe such an approach offers benefits over a strictly organ-based approach. 

If hematocolpos is undiagnosed, serious complications can include ruptured hematosalpinx, urinary tract infections, obstructive uropathy, and future reproductive issues, including increased risk of ectopic pregnancy [14,16]. Performing a genitourinary exam depends on clinical suspicion. Failure to perform a genitourinary exam can result in further delay of diagnosis. Once suspected, a gynecologist should be consulted. In the community setting, this can mean referral and potential transfer to a larger facility.

This work was supported by prior case reports describing the use of POCUS for pediatric abdominal pain [17].

Limitations of this work include the inherent subjectivity of POCUS, where interpretation of ultrasound images can vary between operators.

## Conclusions

Abdominal pain is a common presenting complaint in the ED, particularly among pediatric patients. POCUS is widely available and lacks exposure to ionizing radiation. We propose early incorporation of POCUS in the evaluation of pediatric abdominal pain to decrease diagnostic errors and improve accuracy. We favor a syndromic sonographic approach that is not limited to a single organ or organ system but one that considers the entire differential diagnosis. This case and others emphasize the value of such an approach. A limitation of POCUS is user experience and competency. Emergency physicians working in all settings, including those caring for pediatric patients, should have access to and be competent in performing a wide range of POCUS applications.
